# Comparative effectiveness of percutaneous coronary intervention strategies for coronary small-vessel disease: a network meta-analysis of randomized trials

**DOI:** 10.1080/07853890.2026.2623329

**Published:** 2026-02-03

**Authors:** Changjiang Deng, Chao Fan, Zhiyan Du, Yixin Xu, Tingting Wu, Ying Pan, Mingming Lv, Zhihui Jiang, Bingxin Bai, ZhiLong Wang, Adilai. Adilijiang, Yingying Zheng, Xiang Xie

**Affiliations:** ^a^ Department of Cardiology, The First Affiliated Hospital of Xinjiang Medical University, Urumqi, China; ^b^ Department of Cardiology, The Southwest Hospital of AMU, Chongqing, China; ^c^ Department of Pathophysiology, Xinjiang Medical University College of Basic Medicine, Urumqi, China

**Keywords:** Coronary small-vessel disease, percutaneous coronary intervention, target lesion revascularization, binary restenosis, myocardial infarction, network meta-analysis

## Abstract

**Background:**

Coronary small-vessel disease (SVD) remains challenging for percutaneous coronary intervention (PCI) because small lumens magnify restenosis and ischemic risk. Multiple devices are available, yet their comparative performance is uncertain. This study evaluated and ranked PCI strategies for SVD.

**Methods:**

A systematic review and network meta-analysis was conducted in accordance with PRISMA. PubMed, Embase, the Cochrane Central Register of Controlled Trials, Web of Science, and Google Scholar were searched from inception to 15 August 2025. Eligible studies were English-language randomized controlled trials enrolling adults with angiographic SVD defined as reference vessel diameter ≤3.0 mm, comparing PCI strategies, and reporting target lesion revascularization (TLR), binary restenosis (BR), or myocardial infarction (MI). A frequentist random-effects network meta-analysis generated odds ratios (ORs) with 95% confidence intervals (CIs) and treatment rankings using the surface under the cumulative ranking curve (SUCRA).

**Results:**

Thirty-nine trials including 14,503 patients met the criteria. For TLR (37 studies; 11,980 patients), the highest SUCRA values were observed with sirolimus-eluting stents (SES 90.1%), zotarolimus-eluting stents (ZES 83.9%), and everolimus-eluting stents (EES 82.2%). For BR (32; 6,468), SES, ZES, and paclitaxel-coated balloons (DCB-PTX) ranked highest (95.0%, 80.0%, and 78.3%). For MI (37; 11,602), SES, DCB-PTX, and ZES ranked highest (79.0%, 78.7%, and 68.5%). Representative effects showed SES reduced TLR versus bare-metal stents (BMS) (OR, 0.25; 95% CI, 0.15–0.43) and MI versus BMS (OR, 0.41; 95% CI, 0.21–0.79). Conventional approaches such as BMS, plain old balloon angioplasty (POBA), and gold-plated balloon angioplasty (GPBA) ranked lowest across outcomes.

**Conclusion:**

SES provides the most consistent clinical benefit for coronary SVD. ZES, EES, and DCB-PTX are effective alternatives in selected settings, whereas BMS, POBA, and GPBA are less effective. These findings offer comparative evidence to guide device selection in SVD.

## 1. Introduction

Coronary heart disease (CHD) arises from endothelial injury and lipid deposition that led to the formation of atherosclerotic plaques, subsequent ulceration or rupture, thrombosis, and progressive luminal narrowing or occlusion, ultimately resulting in myocardial ischemia, hypoxia, and necrosis [1]. CHD remains one of the leading causes of morbidity and mortality worldwide. In Western countries, the prevalence in adults aged 20 years or older is reported to be as high as 7.2%, and the global burden continues to increase with population aging [2]. Among its manifestations, coronary small vessel disease (SVD) represents a challenging subset with distinct pathophysiological and therapeutic features. It is most commonly defined angiographically as a reference vessel diameter of 3.0 mm or less, and is frequently observed in patients with diabetes mellitus and chronic kidney disease [3]. Lesions are usually located in mid-to-distal segments and often show diffuse involvement and long lesion length. Because of the inherently small lumen size, the tolerance for neointimal hyperplasia is reduced, predisposing these vessels to higher rates of restenosis and adverse clinical events after revascularization [4]. Epidemiological studies estimate that small vessel disease accounts for 30%–76% of lesions in patients undergoing percutaneous coronary intervention (PCI), underscoring its substantial global prevalence and associated health burden [5]. The high incidence of restenosis and repeat revascularization highlights the need for optimized management strategies.

Historically, coronary artery bypass grafting (CABG) was the predominant method of revascularization, but its invasive nature, perioperative risks, and concerns regarding accelerated atherosclerosis of graft vessels limited its widespread application in small vessel disease [6]. The advent of PCI provided a less invasive alternative, though early balloon angioplasty and bare-metal stents (BMS) were still limited by high restenosis and reintervention rates [7,8]. The introduction of drug-eluting stents (DES) represented a major breakthrough, with first-generation sirolimus-eluting stents showing significant inhibition of smooth muscle proliferation. Second-generation DES, such as everolimus- and zotarolimus-eluting stents, incorporated thinner struts, improved alloys, and more biocompatible polymers to enhance deliverability and long-term safety. Third-generation stents with biodegradable polymers were designed to further reduce chronic inflammatory reactions and late restenosis [9]. In parallel, paclitaxel-eluting stents offered an alternative antimitotic mechanism, and drug-coated balloons (DCBs) emerged as a non-stent-based approach that delivers antiproliferative drugs without leaving a permanent implant [10]. Although DCBs have shown efficacy in inhibiting neointimal proliferation, some studies such as PICCOLETO reported higher restenosis rates, whereas others including BASKET-SMALL 2 demonstrated comparable outcomes with optimal lesion preparation [11,12]. More recently, bioresorbable vascular scaffolds (BVS) were developed to provide temporary vessel support and restore vasomotion; however, trials have reported higher event rates in small vessels, raising safety concerns [13].

A growing body of clinical research has evaluated a wide range of devices for small vessel disease, including balloon angioplasty, BMS, multiple generations of DES, paclitaxel-eluting stents, DCBs, and BVS [14,15]. However, previous meta-analyses have reported inconsistent findings regarding the comparative performance of these interventions. While several studies favored second-generation DES, others found comparable efficacy between DES and DCBs, particularly in short and simple lesions, leading to uncertainty in clinical interpretation [16,17]. Moreover, earlier analyses often included limited device types and excluded newer ultrathin-strut DES and bioresorbable platforms, reducing their relevance to contemporary practice. Many studies on SVD are also derived from subgroup analyses rather than dedicated randomized trials, limiting the generalizability of their conclusions.

Given these uncertainties, a comprehensive comparative evaluation of revascularization strategies for coronary SVD is warranted. Network meta-analysis allows integration of both direct and indirect evidence across multiple interventions within a single framework and enables ranking of treatment options by efficacy and safety [18]. By synthesizing the totality of randomized evidence, this approach can generate clinically meaningful insights beyond those provided by traditional pairwise comparisons. The present study was therefore designed to systematically compare and rank different PCI modalities for coronary SVD, aiming to identify the optimal treatment strategy and provide comprehensive evidence to guide clinical practice and support future clinical guidance.

## 2. Methods

This systematic review and network meta-analysis was conducted and reported in accordance with the 2020 Preferred Reporting Items for Systematic Reviews and Meta-Analyses (PRISMA) statement and its extension for network meta-analyses of health care interventions [19,20]. The protocol was registered in PROSPERO (CRD420251140071). As the analysis used only data from previously published studies, institutional review board approval and informed consent were not required.

### 2.1. Data sources and search strategy

A comprehensive literature search was performed in PubMed, Embase, the Cochrane Central Register of Controlled Trials (CENTRAL), Web of Science, and Google Scholar from inception to 15 August 2025. The search strategy combined controlled vocabulary (MeSH and Emtree terms) and free-text keywords related to coronary small vessel disease (SVD) (e.g. ‘coronary microvascular dysfunction,’ ‘coronary small vessel disease’), percutaneous coronary intervention (PCI) techniques (e.g. ‘stents,’ ‘balloon angioplasty’), and randomized controlled trials. Boolean operators ‘AND’ and ‘OR’ were used to combine terms appropriately. The complete search strategies for each database are provided in Table S1. To minimize the risk of missing eligible studies, the reference lists of all included articles and relevant systematic reviews published in the past five years were manually screened.

Two reviewers independently screened titles and abstracts, followed by full-text assessment of potentially relevant articles. Discrepancies were resolved through discussion or, if necessary, adjudication by a third reviewer.

### 2.2. Eligibility criteria

Studies were included if they met the following criteria: (a) the population comprised adult patients diagnosed with coronary small-vessel disease, defined angiographically as a reference vessel diameter ≤3.0 mm, consistent with definitions used in several prior meta-analyses. We acknowledge that some studies define small vessels more stringently as <2.5 mm, and this variability in criteria was considered during the analysis [21]; (b) the intervention involved percutaneous coronary intervention (PCI) using any eligible device type or technique, including bare-metal stents (BMS), drug-eluting stents (DES; such as everolimus-eluting stents [EES], sirolimus-eluting stents [SES], paclitaxel-eluting stents [PES], or zotarolimus-eluting stents [ZES]), drug-coated balloons (DCB; including DCB-BMS and paclitaxel-coated balloons [DCB-PTX]), cutting balloon angioplasty (CBA), conventional balloon angioplasty (POBA), or other PCI devices such as gold-plated balloon angioplasty (GPBA); (c) the comparator was another eligible PCI strategy, including balloon-based interventions (POBA, CBA, GPBA), bare-metal stents (BMS), or alternative drug-eluting or drug-coated technologies (e.g. DCB or DES of different generations), or, where applicable, conventional medical management without PCI; (d) the study reported at least one of the predefined clinical outcomes—target lesion revascularization (TLR), binary restenosis (BR), or myocardial infarction (MI)—at the longest available follow-up; (e) the design was a randomized controlled trial (RCT) published in English with sufficient quantitative data for effect size estimation.

Studies were excluded if they (a) involved populations with large-vessel coronary artery disease, bypass graft disease, or acute coronary syndromes without isolated small-vessel involvement; (b) evaluated interventions or comparators not involving PCI, or where both arms received the identical PCI strategy; (c) did not provide clear descriptions of the intervention techniques; (d) lacked relevant outcome data or sufficient numerical information for analysis, with no author response to at least four data requests over six weeks; (e) were non-randomized studies, observational designs, case series, case reports, conference abstracts, protocols, or duplicate publications.

### 2.3. Data extraction

All eligible studies were managed in EndNote X9 to remove duplicates. Data extraction was independently conducted by two reviewers using a standardized form (Table S2), which included bibliographic information (authors, year, and journal), study characteristics (sample size, setting, inclusion and exclusion criteria), patient demographics and clinical characteristics (age, sex, comorbidities, lesion characteristics), intervention and comparator details, follow-up duration, and outcome measures. For continuous variables, means and standard deviations were extracted directly or calculated from medians and interquartile ranges using methods recommended in the Cochrane Handbook [22]. When essential data were unavailable, study authors were contacted up to four times over a six-week period; if no response was received, the study was excluded from that specific outcome analysis. For studies with multiple arms using the same PCI category, data were combined according to Cochrane guidelines.

### 2.4. Risk-of-bias assessment

The risk of bias for each included trial was assessed at the study level using the revised Cochrane risk-of-bias tool for randomized trials (RoB 2), considering the domains of randomization process, deviations from intended interventions, missing outcome data, measurement of the outcome, and selection of the reported result [23]. Disagreements between reviewers were resolved by consensus or, when necessary, by a third reviewer.

### 2.5. Data coding

PCI strategies were coded according to the categories used in the network meta-analysis, namely BMS, CBA, conventional cutting stent (CCS), DCB-BMS, DCB-PTX, EES, GPBA, PES, POBA, SES, and ZES, with each trial arm assigned to one of these categories.

### 2.6. Statistical analysis

All statistical analyses were performed using Stata version 17.0 (StataCorp LLC, College Station, TX, USA) and R version 4.3.2 with the network and mvmeta packages. A random-effects network meta-analysis was conducted within a frequentist framework to integrate direct and indirect evidence and compare all PCI strategies simultaneously, with random-effects modeling chosen a priori to account for anticipated clinical and methodological heterogeneity. Effect estimates for all outcomes were expressed as odds ratios (ORs) with 95% confidence intervals (CIs). Heterogeneity was assessed using the I^2^ statistic, with values of 25%, 50%, and 75% representing low, moderate, and high heterogeneity, respectively. Global inconsistency was evaluated using the design-by-treatment interaction model, and local inconsistency was examined using the node-splitting method. Treatment ranking was performed using the surface under the cumulative ranking curve (SUCRA) and mean rank values, with higher SUCRA values indicating a greater likelihood of being the most effective intervention. Predictive interval plots were generated to illustrate the dispersion of treatment effects accounting for heterogeneity.

To explore potential structural confounding, a random-effects meta-regression was conducted using stent strut thickness (μm) as a continuous covariate, with coefficients and odds ratios expressed per 10-μm increase. Between-study variance (τ^2^) before and after adjustment was compared to evaluate model fit and heterogeneity reduction. Sensitivity analyses included excluding trials at high risk of bias, restricting analyses to trials with follow-up duration of at least 12 months, and applying fixed-effect models. Publication bias and small-study effects were assessed using comparison-adjusted funnel plots and Egger’s test, with a *p* value of less than 0.05 considered indicative of bias [24]. All statistical tests were two-sided, and *p* values below 0.05 were regarded as statistically significant.

## 3. Results

### 3.1. Characteristics of included studies

**Figure 1.**
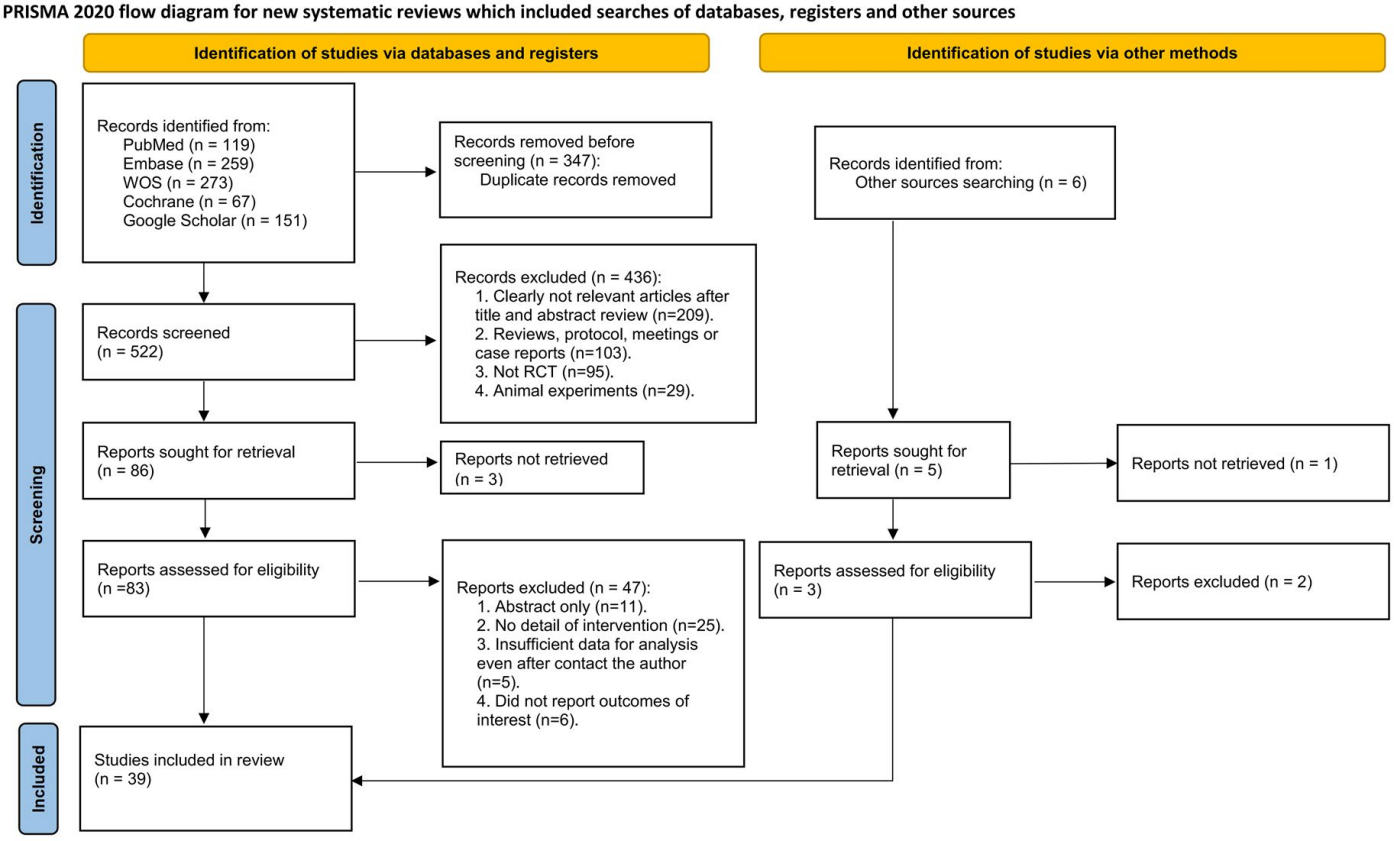
PRISMA Flow diagram of the search process for studies. Flow diagram showing identification, screening, eligibility assessment, and inclusion of randomized controlled trials in accordance with PRISMA 2020.

The initial electronic search identified 869 records. After removal of 347 duplicates, 522 titles and abstracts were screened, 436 records were excluded at this stage, and 86 full texts were assessed for eligibility; ultimately, 39 randomized controlled trials comprising 14,503 patients with coronary small vessel disease were included in the systematic review and network meta-analysis (Figure 1) [12,25–34]. The trials were published between 1998 and 2024, with a median publication year of 2009. Sample sizes ranged from 41 to 2008 participants, with a median of 230 per study. The mean age of participants across trials ranged from 59 to 69 years, with a median of 64.2 years. With respect to procedural strategies, 16 trials evaluated bare-metal stents (BMS), 1 evaluated cutting balloon angioplasty (CBA), 1 evaluated conventional cutting stents (CCS), 3 evaluated drug-coated balloon plus bare-metal stent (DCB-BMS), 12 evaluated paclitaxel-coated balloons (DCB-PTX), 10 evaluated everolimus-eluting stents (EES), 8 evaluated gold-plated balloon angioplasty (GPBA), 7 evaluated paclitaxel-eluting stents (PES), 9 evaluated plain old balloon angioplasty (POBA), 8 evaluated sirolimus-eluting stents (SES), and 4 evaluated zotarolimus-eluting stents (ZES). To provide additional clarity on event distribution, the absolute frequencies of all primary endpoints (TLR, BR, and MI) extracted from each trial have been summarized in Table S2. Detailed study characteristics are provided in Table S2.

### 3.2. Results of the network meta-analysis

#### 3.2.1. Target lesion revascularization (TLR)

**Figure 2.**
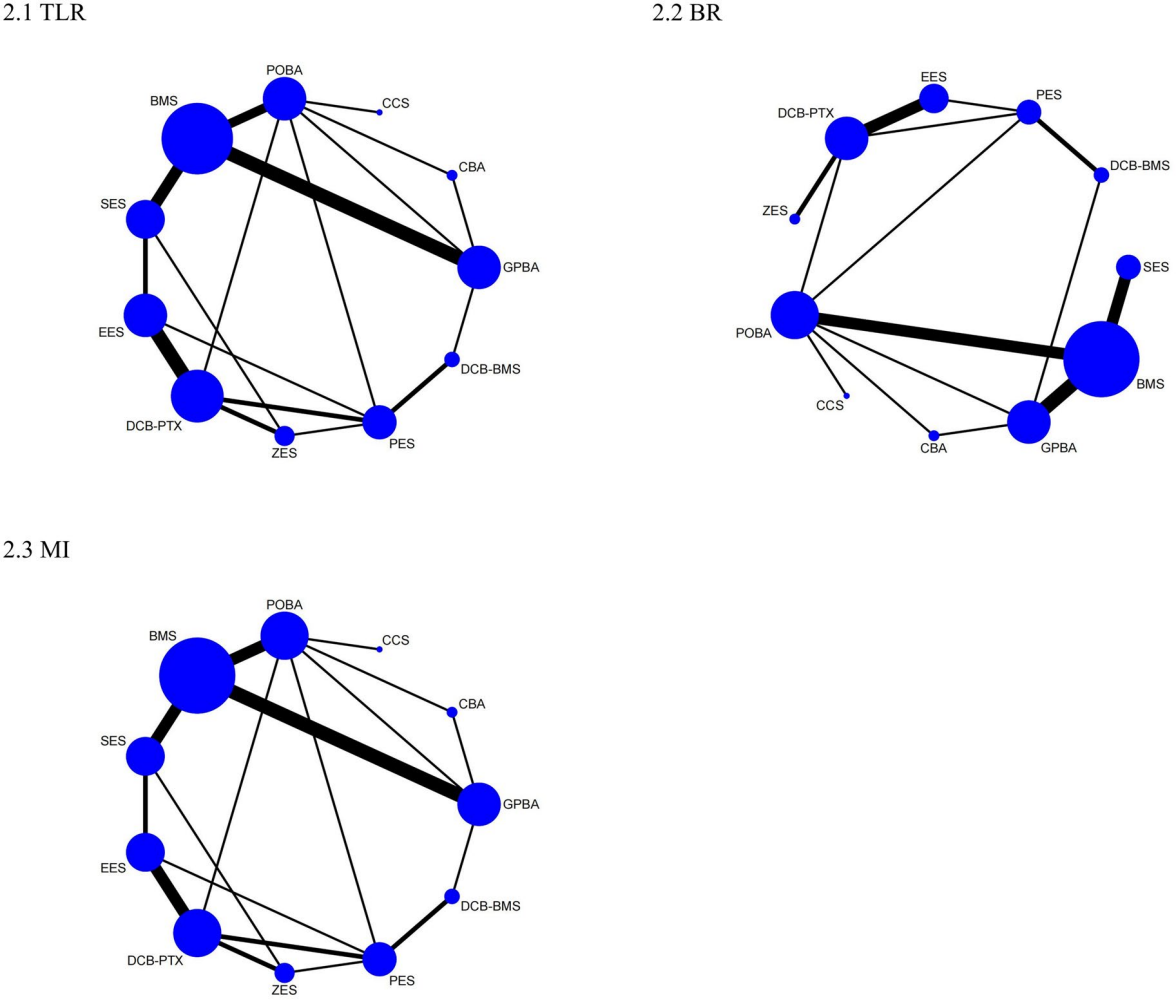
Network plot comparing efficacy outcomes: (A) TLR; (B) BR; (C) MI. Nodes represent PCI strategies (size proportional to sample size), and lines indicate direct comparisons (thickness proportional to number of trials) for (A) TLR, (B) BR, and (C) MI.

For TLR, 37 studies with 11,980 patients contributed to the network. The network geometry and the distribution of direct comparisons are presented in Figure 2A. The SUCRA rankings (Figure S2) indicated that SES (90.1%), ZES (83.9%), and EES (82.2%) were the top strategies for reducing TLR, with GPBA ranked lowest (15.3%). SES significantly reduced TLR compared to GPBA, CCS, POBA, BMS, DCB-BMS, and PES. ZES and EES also significantly reduced TLR compared to GPBA, CCS, POBA, and BMS. DCB-PTX showed significant reductions in TLR compared to GPBA, CCS, POBA, BMS, and PES, as summarized in Table 1.

**Table 1.** League table of TLR outcomes in patients.

	SES (90.1%)	ZES (83.9%)	EES (82.2%)	DCB-PTX (78.1%)	PES (48.0%)	CBA (44.3%)	DCB-BMS (39.1%)	BMS (30.5%)	POBA (21.5%)	CCS (17.0%)	GPBA (15.3%)	
SES (90.1%)	–	1.12	1.18	1.27	**2.70**	2.94	**3.23**	**4.00**	**4.55**	**5.88**	**5.00**	
ZES (83.9%)	0.89	–	1.05	1.14	2.38	2.63	2.86	**3.57**	**4.00**	**5.26**	**4.55**	
EES (82.2%)	0.85	0.95	–	1.08	**2.27**	2.50	2.70	**3.33**	**3.85**	**5.00**	**4.35**	
DCB-PTX (78.1%)	0.79	0.88	0.93	–	**2.13**	2.27	2.56	**3.13**	**3.57**	**4.55**	**4.00**	
PES (48.0%)	**0.37**	0.42	**0.44**	**0.47**	–	1.08	1.19	1.47	1.69	2.17	1.85	
CBA (44.3%)	0.34	0.38	0.4	0.44	0.93	–	1.10	1.37	1.56	2.00	1.72	
DCB-BMS (39.1%)	**0.31**	0.35	0.37	0.39	0.84	0.91	–	1.23	1.41	1.82	1.56	
BMS (30.5%)	**0.25**	**0.28**	**0.3**	**0.32**	0.68	0.73	0.81	–	1.14	1.47	1.27	
POBA (21.5%)	**0.22**	**0.25**	**0.26**	**0.28**	0.59	0.64	0.71	0.88	–	1.28	1.10	
CCS (17.0%)	**0.17**	**0.19**	**0.2**	**0.22**	0.46	0.5	0.55	0.68	0.78	–	0.85	
GPBA (15.3%)	**0.2**	**0.22**	**0.23**	**0.25**	0.54	0.58	0.64	0.79	0.91	1.17	–	

This table presents odds ratios (ORs) for target lesion revascularization (TLR) comparing PCI strategies, with values >1.0 indicating a higher TLR risk for the row-defining treatment and values <1.0 indicating a lower risk. Bold values denote statistically significant comparisons (95% confidence intervals not crossing 1.0). Green and red shading indicate statistically significant risk reduction or increase, respectively, favoring the row or column treatment. Color intensity reflects the magnitude of the effect size, whereas gray shading indicates non-significant or insufficiently informed comparisons. SUCRA values are shown in parentheses to indicate relative treatment ranking.

In the primary (unadjusted) network meta-analysis, pairwise comparisons among contemporary DES (SES, EES, and ZES) were not statistically significant, although the point estimates showed modest numerical gradients consistent with the SUCRA ordering. After adjusting for stent strut thickness in the meta-regression model, the effect estimates among new-generation DES (SES, EES, and ZES) remained directionally consistent, and the numerical gradients were modestly attenuated toward the null, while DES-DES comparisons remained non-significant, (Table S4).

#### 3.2.2. Binary restenosis (BR)

For BR, 32 studies with 6,468 patients contributed to the network. The direct evidence and sample dispersion are depicted in Figure 2B. SUCRA rankings (Figure S2) showed SES (95.0%), ZES (80.0%), and DCB-PTX (78.3%) as the most effective treatments for lowering BR, with GPBA ranked lowest (8.8%). SES significantly reduced BR compared to GPBA, POBA, BMS, CBA, DCB-BMS, and PES. ZES and DCB-PTX also showed significant reductions in BR compared to GPBA, POBA, and BMS. EES significantly reduced BR compared to GPBA, POBA, and BMS. CCS and PES also showed significant reductions in BR compared to GPBA and POBA. Full comparisons are detailed in Table 2.

**Table 2.** League table of BR outcomes in patients.

	SES (95.0%)	ZES (80.0%)	DCB-PTX (78.3%)	EES (74.3%)	CCS (61.6%)	PES (51.0%)	DCB-BMS (34.5%)	CBA (28.1%)	BMS (26.3%)	POBA (11.9%)	GPBA (8.8%)	
SES (95.0%)	–	1.85	2.00	2.22	3.23	**4.76**	**7.14**	**8.33**	**9.09**	**11.11**	**12.50**	
ZES (80.0%)	0.54	–	1.09	1.20	1.75	2.50	3.85	4.55	**5.00**	**6.25**	**6.67**	
DCB-PTX (78.3%)	0.50	0.92	–	1.10	1.61	2.33	**3.57**	**4.35**	**4.55**	**5.88**	**6.25**	
EES (74.3%)	0.45	0.83	0.91	–	1.47	2.08	3.23	3.85	**4.17**	**5.26**	**5.56**	
CCS (61.6%)	0.31	0.57	0.62	0.68	–	1.43	2.17	2.63	2.86	**3.57**	**3.85**	
PES (51.0%)	**0.21**	0.40	0.43	0.48	0.70	–	1.54	1.85	2.00	**2.50**	**2.70**	
DCB-BMS (34.5%)	**0.14**	0.26	**0.28**	0.31	0.46	0.65	–	1.20	1.30	1.64	1.75	
CBA (28.1%)	**0.12**	0.22	**0.23**	0.26	0.38	0.54	0.83	–	1.08	1.37	1.45	
BMS (26.3%)	**0.11**	**0.20**	**0.22**	**0.24**	0.35	0.50	0.77	0.93	–	1.27	1.35	
POBA (11.9%)	**0.09**	**0.16**	**0.17**	**0.19**	**0.28**	**0.40**	0.61	0.73	0.79	–	1.06	
GPBA (8.8%)	**0.08**	**0.15**	**0.16**	**0.18**	**0.26**	**0.37**	0.57	0.69	0.74	0.94	–	

This table presents odds ratios (ORs) for binary restenosis (BR) comparing PCI strategies, with values >1.0 indicating a higher BR risk for the row-defining treatment and values <1.0 indicating a lower risk. Bold values denote statistically significant comparisons (95% confidence intervals not crossing 1.0). Green and red shading indicate statistically significant BR risk reduction or increase, respectively, favoring the row or column treatment, while color intensity reflects the magnitude of the effect size. Gray shading denotes non-significant or insufficiently informed comparisons. SUCRA values are shown in parentheses to indicate relative treatment ranking.

In the strut thickness meta-regression, thicker stents were associated with higher BR risk (per 10 μm: OR 1.09, 95% CI 1.03–1.15), but this adjustment produced only small shifts in the DES point estimates and did not materially change the ordering of contemporary DES, indicating that structural design may partially mediate but does not fully explain their differences.

#### 3.2.3. Myocardial infarction (MI)

For MI, 37 studies with 11,602 patients contributed data. The network structure and distribution of direct comparisons are shown in Figure 2C. SUCRA rankings (Figure S2) indicated that SES (79.0%), DCB-PTX (78.7%), and ZES (68.5%) were the top treatments for reducing MI, with GPBA ranked lowest (14.6%). SES significantly reduced MI compared to GPBA, POBA, and BMS. DCB-PTX also significantly reduced MI compared to GPBA, POBA, and BMS. EES significantly reduced MI compared to GPBA, see in Table 3.

**Table 3.** League table of MI outcomes in patients.

	SES (79.0%)	DCB-PTX (78.7%)	ZES (69.5%)	EES (66.4%)	CCS (60.3%)	DCM-BMS (53.9%)	CBA (45.2%)	PES (34.3%)	BMS (26.2%)	POBA (22.8%)	GPBA (14.6%)	
SES (79.0%)	–	0.96	1.14	1.19	1.19	1.52	1.82	2.08	**2.44**	**2.56**	**2.94**	
DCB-PTX (78.7%)	1.04	–	1.18	1.23	1.23	1.59	1.89	2.17	**2.50**	**2.63**	**3.03**	
ZES (69.5%)	0.88	0.85	–	1.04	1.04	1.33	1.59	1.85	2.13	2.22	2.56	
EES (66.4%)	0.84	0.81	0.96	–	1.00	1.28	1.52	1.75	2.04	2.13	**2.44**	
CCS (60.3%)	0.84	0.81	0.96	1.00	–	1.28	1.52	1.75	2.04	2.13	2.44	
DCM-BMS (53.9%)	0.66	0.63	0.75	0.78	0.78	–	1.19	1.37	1.59	1.67	1.92	
CBA (45.2%)	0.55	0.53	0.63	0.66	0.66	0.84	–	1.16	1.35	1.41	1.61	
PES (34.3%)	0.48	0.46	0.54	0.57	0.57	0.73	0.86	–	1.15	1.22	1.39	
BMS (26.2%)	**0.41**	**0.40**	0.47	0.49	0.49	0.63	0.74	0.87	–	1.05	1.20	
POBA (22.8%)	**0.39**	**0.38**	0.45	0.47	0.47	0.60	0.71	0.82	0.95	–	1.14	
GPBA (14.6%)	**0.34**	**0.33**	0.39	**0.41**	0.41	0.52	0.62	0.72	0.83	0.88	–	

This table presents odds ratios (ORs) for myocardial infarction (MI) comparing PCI strategies, with values >1.0 indicating a higher MI risk for the row-defining treatment and values <1.0 indicating a lower risk. Bold values denote statistically significant comparisons (95% confidence intervals not crossing 1.0). Green and red shading indicate statistically significant MI risk reduction or increase, respectively, favoring the row or column treatment, while color intensity reflects the magnitude of the effect size. Gray shading denotes non-significant or insufficiently informed comparisons. SUCRA values are shown in parentheses to indicate relative treatment ranking.

The association between strut thickness and MI was not statistically significant (*p* = 0.11), suggesting that ischemic outcomes were less influenced by structural parameters compared with restenosis-related endpoints (Table S4).

### 3.3. Risk of bias and publication bias

Across the 39 trials, the overall risk-of-bias judgment was low in 20 studies and raised some concerns in 19 studies. For the randomization process, 35 trials were judged to be at low risk and 4 at some concerns; for deviations from intended interventions, 30 were at low risk and 9 at some concerns; for missing outcome data, 24 were at low risk and 15 at some concerns; for measurement of the outcome, all 39 trials were at low risk; and for selection of the reported result, all 39 trials were at low risk (Table S3).

Potential publication bias was explored with funnel plots (Figure S1). The scatter around the vertical axis showed varying degrees of asymmetry, suggesting possible small-study effects, with Figure S1.1 through Figure S1.3 demonstrating some asymmetry on visual inspection; however, Egger’s tests for all outcomes yielded p values greater than 0.05, indicating no statistically significant evidence of publication bias in the overall analyses.

## 4. Discussion

In this network meta-analysis of 39 randomized controlled trials including 14,503 patients with coronary small-vessel disease, we systematically compared the effectiveness and safety of contemporary percutaneous coronary intervention strategies. Among new-generation drug-eluting stents, SES achieved the highest SUCRA rankings across major endpoints; however, differences among SES, ZES, and EES were not statistically significant, indicating broadly comparable clinical performance. These limus-eluting stents consistently outperformed older-generation DES, BMS, and POBA, underscoring the overall superiority of modern DES in small-vessel disease. ZES, EES, and DCB-PTX demonstrated advantages in selected outcomes, with ZES and EES reducing TLR and BR, and DCB-PTX showing comparable efficacy to DES for BR and MI while avoiding permanent implantation. In contrast, conventional strategies, including BMS, POBA, GPBA, and first-generation DES, were uniformly inferior across endpoints. Collectively, these findings define a clear performance hierarchy in small-vessel PCI, in which new-generation DES show similar effectiveness and DCB-PTX represents a viable non-stent alternative in appropriately selected patients.

The current findings are generally consistent with previous meta-analyses in this field. Siontis et al. similarly demonstrated that new-generation DES outperform older stents and balloon angioplasty, while showing no significant efficacy differences among SES, ZES, and EES [35]. In line with more recent evidence, our study also supports the comparable outcomes of DCB and DES in appropriately prepared small-vessel lesions [21]. Compared with these earlier studies, the present analysis included a larger number of RCTs and incorporated more recent data, allowing a more comprehensive evaluation of contemporary devices. While SES achieved numerically superior rankings, our meta-regression indicated that this pattern was partly explained by differences in stent strut thickness. Thicker-strut platforms were associated with increased risks of TLR and BR, whereas the effect was not significant for MI. Among SES, EES, and ZES, adjustment mainly shifted point estimates modestly toward unity while comparisons remained non-significant, suggesting a contribution of platform structure alongside drug type.

SES are polymer-coated coronary stents that elute sirolimus, a potent inhibitor of the mTOR pathway that exerts cytostatic effects on vascular smooth-muscle cells and curbs extracellular matrix deposition. As the mechanistic rationale for limus-eluting platforms is well established, we focus on the comparative findings: within this network meta-analysis, SES demonstrated favorable outcomes across TLR, BR, and MI, although these advantages should be interpreted as part of an overall trend rather than conclusive statistical superiority. These findings corroborate prior randomized evidence showing that sirolimus-family DES outperform earlier-generation stents, BMS, and balloon-only approaches in complex lesions, while refining previous observations by situating all devices within a unified comparative hierarchy [36,37].

Although SES achieved the numerically top ranking, real-world decision-making must also account for considerations such as device cost, regional availability, deliverability in tortuous anatomy, and institutional familiarity [38]. Against this pragmatic backdrop, ZES, EES, and DCB-PTX remain credible alternatives with clinically relevant advantages in specific settings. ZES and EES achieved favorable rankings for reducing repeat revascularization and restenosis, while DCB-PTX performed comparably to DES in preventing restenosis and ischemic events without leaving a permanent implant. These findings are consistent with prior pooled studies, further supporting the interchangeability of new-generation DES and the clinical utility of DCB when long-term stent implantation is undesirable.

Biological and engineering attributes plausibly account for the observed performance of these alternatives. ZES and EES combine limus elution with thin metal scaffolds and biocompatible polymers, supporting durable antirestenotic efficacy in SVD [39–41]. DCB-PTX delivers antiproliferative drug without a permanent implant and can approach DES performance when lesion preparation is optimal and bailout stenting is applied when indicated [21]. These attributes likely underlie the favorable rankings of ZES/EES for restenosis-related endpoints and support DCB-PTX as a pragmatic non-stent option in selected anatomies.

Consistent with the superiority of SES and the next best options (ZES, EES, and DCB-PTX) shown in our results, the performance of legacy strategies was uniformly weaker when the same outcomes were considered. Across the network, BMS, POBA, GPBA, and several first-generation DES occupied the lower ranks for TLR, BR, and MI, with higher risks than contemporary limus-eluting platforms and DCB-PTX. This pattern appeared in both direct and indirect contrasts, which suggests a reproducible class effect rather than study-specific noise. The findings align with prior randomized trials and pooled analyses that reported better clinical and angiographic outcomes with modern DES than with BMS or balloon angioplasty, and they help reconcile earlier reports of acceptable results with balloon-only therapy by indicating that those results likely reflect short, simple lesions treated with meticulous preparation in carefully selected patients rather than a general advantage in SVD [42]. The likely explanation is straightforward: POBA is prone to recoil and dissection, BMS lacks antiproliferative effect, and GPBA adds injury without suppressing neointimal growth; earlier DES were limited by thicker struts and less biocompatible polymers [43].

This analysis provides a practical framework for device selection in coronary SVD. Taken together, the rankings and pairwise estimates support SES as the default PCI strategy when deliverability is feasible and cost and availability are not prohibitive. In patients with very small reference diameters, diffuse disease, or a preference to avoid a permanent implant or prolonged dual antiplatelet therapy, DCB-PTX is a reasonable alternative provided that lesion preparation is meticulous and bailout criteria are respected. ZES and EES performed well for prevention of reintervention and restenosis and are suitable options where SES is unavailable or when operator familiarity, anatomical constraints, or institutional formulary considerations favor these platforms. The inferiority of BMS, POBA, GPBA and several first-generation DES across outcomes supports their restriction to bailout or resource-constrained scenarios. These conclusions align with the pathophysiology of SVD, where small lumens magnify the clinical impact of neointimal growth and late events. Given that a vessel with a reference diameter of 2.25 mm differs substantially in biomechanical and clinical behavior from one of 3.0 mm, the variation in vessel size among studies could have influenced outcomes. Most included trials did not report vessel-diameter–specific data, precluding stratified analysis; future investigations should address this aspect to better define device performance across vessel calibers. Moreover, the included trials encompassed a range of clinical settings, including both stable angina and acute coronary syndromes, although few studies reported stratified outcomes by indication. The influence of these different clinical contexts—particularly primary PCI in acute myocardial infarction—on device performance could not be evaluated in this analysis and warrants further investigation in future stratified or patient-level studies. In practice, the hierarchy observed here can complement intravascular imaging guidance and careful lesion preparation to reduce target lesion failure. More broadly, the signal for lower myocardial infarction with the top-ranked strategies indicates that optimizing device choice in SVD has consequences beyond angiographic success and may translate into better long-term clinical outcomes.

This study has several strengths. It synthesizes a large body of randomized evidence using a prespecified random-effects network meta-analysis that integrates direct and indirect comparisons, formally evaluates heterogeneity and inconsistency, and applies transparent, PRISMA-compliant methods focused on clinically meaningful endpoints. Limitations should also be acknowledged. Heterogeneity in SVD definitions, procedural techniques, device generations, and follow-up duration across trials introduces uncertainty, and some network nodes were informed by few studies. Device-specific structural factors, such as strut thickness, may partly confound outcomes and could not be fully isolated using study-level data. Analyses based on aggregate rather than individual patient data precluded adjustment for important modifiers, including reference vessel diameter and comorbidities. In addition, inclusion of English-language trials only and variable follow-up durations may introduce reporting bias. These considerations warrant cautious interpretation of SUCRA rankings but do not detract from the overall performance hierarchy and its relevance for clinical practice.

## 5. Conclusion

In this network meta-analysis of contemporary interventional strategies for coronary small vessel disease, new-generation drug-eluting stents (SES, ZES, and EES) demonstrated comparable and favorable outcomes across all major endpoints, with SES ranking highest numerically but without statistically significant differences among these stents. DCB-PTX also showed favorable results and may serve as an effective alternative when stent implantation is not feasible. In contrast, conventional approaches such as BMS, POBA, GPBA, and certain first-generation DES were associated with poorer clinical outcomes, underscoring their limited role in current practice. These findings provide robust comparative evidence to guide clinical decision-making, emphasizing the need to prioritize new-generation DES and, where appropriate, DCB strategies for patients with SVD. Further head-to-head randomized trials are warranted to validate these results, particularly in high-risk subgroups, and to optimize revascularization strategies for this challenging population.

## Supplementary Material

PRISMA_2020_checklist.pdf

Revised Supplementary-updated.docx

## Data Availability

The data that support the findings of this study are available from the corresponding author upon reasonable request. In line with the Taylor & Francis ‘Share Upon Reasonable Request’ policy, de-identified data and relevant materials will be made available to qualified researchers upon reasonable request. Certain restrictions (e.g. ethical, privacy, or security concerns) may apply.
