# Supplementary material for: Comparative effectiveness of percutaneous coronary intervention strategies for coronary small-vessel disease: a network meta-analysis of randomized trials
Source: Ann Med. 2026 Feb 3;58(1):2623329. doi: 10.1080/07853890.2026.2623329 (PMC12872083; doi:10.1080/07853890.2026.2623329)
Supplement: Revised Supplementary-updated.docx [file IANN_A_2623329_SM4129.docx]

**Supplementary**

Table of Contents:

**[Supplementary Table 1: Search Strategy 2](#_Toc159076731)**

**Supplementary** [Table 2: Characteristics of studies and subjects included in the review 4](#_Toc159076743)

**Supplementary** [Table 3: Risk of Bias](#_Toc159076747) 14

**Supplementary** [Table 4: Meta-regression with strut thickness](#_Toc159076747) 17

**Supplementary** [Figure 1: Funnel Plots of Publication Bias](#_Toc159076747) 18

**Supplementary** [Figure 2: SUCRA probability ranking plot](#_Toc159076747) 21

# Table S1: Search Strategy

***Search Strategy:***

| #8 | Search: (((((("Coronary Microvascular Dysfunction"[Title/Abstract]) OR ("microvascular angina"[Title/Abstract])) OR ("coronary small vessel disease"[Title/Abstract])) OR ("small coronary vessel"[Title/Abstract])) OR ("small coronary artery"[Title/Abstract])) AND (((((((((((Stents[MeSH Terms]) OR (stent*[Title/Abstract])) OR ("Bare Metal Stent"[Title/Abstract])) OR ("Drug-Eluting Stent"[Title/Abstract])) OR ("Drug-Coated Balloon"[Title/Abstract])) OR ("Percutaneous Transluminal Balloon Angioplasty"[Title/Abstract])) OR ("balloon angioplasty"[Title/Abstract])) OR (Angioplasty, Balloon, Coronary[MeSH Terms])) OR ("percutaneous coronary intervention"[Title/Abstract])) OR (angioplasty[Title/Abstract])) OR ("transluminal coronary angioplasty"[Title/Abstract]))) AND ((randomized controlled trial[pt] OR controlled clinical trial[pt] OR randomized[tiab] OR placebo[tiab] OR drug therapy[sh] OR randomly[tiab] OR trial[tiab] OR groups[tiab]) NOT (animals[mh] NOT humans[mh])) |
| --- | --- |
| #7 | Search: (randomized controlled trial[pt] OR controlled clinical trial[pt] OR randomized[tiab] OR placebo[tiab] OR drug therapy[sh] OR randomly[tiab] OR trial[tiab] OR groups[tiab]) NOT (animals[mh] NOT humans[mh]) |
| #6 | Search: ((((((((((Stents[MeSH Terms]) OR (stent*[Title/Abstract])) OR ("Bare Metal Stent"[Title/Abstract])) OR ("Drug-Eluting Stent"[Title/Abstract])) OR ("Drug-Coated Balloon"[Title/Abstract])) OR ("Percutaneous Transluminal Balloon Angioplasty"[Title/Abstract])) OR ("balloon angioplasty"[Title/Abstract])) OR (Angioplasty, Balloon, Coronary[MeSH Terms])) OR ("percutaneous coronary intervention"[Title/Abstract])) OR (angioplasty[Title/Abstract])) OR ("transluminal coronary angioplasty"[Title/Abstract]) |
| #5 | Search: ("Drug-Coated Balloon"[Title/Abstract])) OR ("Percutaneous Transluminal Balloon Angioplasty"[Title/Abstract])) OR ("balloon angioplasty"[Title/Abstract])) OR ("percutaneous coronary intervention"[Title/Abstract])) OR (angioplasty[Title/Abstract])) OR ("transluminal coronary angioplasty"[Title/Abstract]) |
| #4 | Search: Angioplasty, Balloon, Coronary[MeSH Terms] |
| #3 | Search: (stent*[Title/Abstract])) OR ("Bare Metal Stent"[Title/Abstract])) OR ("Drug-Eluting Stent"[Title/Abstract]) |
| #2 | Search: Stents[MeSH Terms] |
| #1 | Search: (((("Coronary Microvascular Dysfunction"[Title/Abstract]) OR ("microvascular angina"[Title/Abstract])) OR ("coronary small vessel disease"[Title/Abstract])) OR ("small coronary vessel"[Title/Abstract])) OR ("small coronary artery"[Title/Abstract]) |

# Table S2: Characteristics of studies and subjects included in the review

| **Study** | **Country/Region** | **Subjects**  **(intervention/ control)** | **Sex (male/female)**  **(intervention/ control)** | **Mean age**  **(intervention/ control)** | **Type of disease** | **Intervention detail** | **Control group** | **Follow-up** | **Outcomes** | **BR** | **TLR** | **MI** |
| --- | --- | --- | --- | --- | --- | --- | --- | --- | --- | --- | --- | --- |
| Fahrni et al. (2020a) | Multicentre | 111 (DCB 47 / DES 64) | 38/9 vs. 45/19 | 68.1 ± 9.4 vs. 66.6 ± 11.3 | Mixed population with stable CAD and ACS | DCB-PTX: SeQuent Please® drug-coated balloon (paclitaxel–iopromide) | EES: Early Taxus Element® (paclitaxel) → Later Xience® (everolimus) | DES 175 d vs. DCB 170 d | BR | 14/66 | 10/51 |  |
| Giannini et al. (2017) | Italy | 182 (DCB 91 / DES 91) | NA | NA | Small vessel coronary artery disease (RVD < 2.8 mm) | DCB-PTX: IN.PACT Falcon® paclitaxel-coated balloon (30–60 s inflation) | EES: Taxus Libertè® (paclitaxel) → later switch to Xience® (everolimus) | 6 months | TLR, BR, MI | 2/32 | 8/32 | 4/91 |
| Jeger et al. (2020b) | Multicentre | 758 (DCB 382 / DES 376) | 87 / 295 vs. 114 / 262 | 67.18 ± 10.33 vs. 68.42 ± 10.32 | De novo small vessel coronary artery disease (diameter ≥2.0 to <3.0 mm; ACS, stable angina, or silent ischemia) | DCB-PTX: paclitaxel-coated SeQuent Please® balloon | EES: everolimus-eluting Xience® (72%) or paclitaxel-eluting Taxus Element® (28%) | 6 months | TLR, BR | 19/382 | 23/376 | 30/382 |
| Kwon et al. (2003) | Korea | 41 (RA 21 / BP 20) | NA | NA | LAD small vessel disease (2.0–2.9 mm diameter) | BMS: Rotational Atherectomy + Bare-Metal Stent | POBA: Balloon Predilation | 6 months | TLR, BR, MI | 6/18 | 5/16 | 8/21 |
| Liu et al. (2024) | China | 247 (DCB 129 / DES 118) | 94 / 35 vs. 82 / 36 | 60.2 ± 9.5 vs. 60.1 ± 9.3 | De novo small vessel coronary artery disease (RVD 2.25–2.75 mm; ACS, stable angina, or silent ischemia) | DCB-PTX: Dissolve DCB (3 mg paclitaxel/mm² + medium chain triglyceride co-solvent) | ZES: Endeavor Resolute zotarolimus-eluting stent | 9 ± 1 months | TLR, BR, MI | 11/117 | 10/98 | 11/129 |
| Kawai et al. (2021) | Japan | 42 (DCB 19 / DES 23) | 15 / 4 vs. 17 / 6 | 69 ± 8 vs. 73 ± 8 | De novo small vessel CAD (reference diameter 2.0–3.0 mm; stable angina or silent ischemia) | DCB-PTX: SeQuent® Please drug-coated balloon (≥30 s inflation) | EES: SYNERGY™ bioresorbable polymer everolimus-eluting stent | 8 months | TLR, BR, MI | 3/19 | 1/22 | 0/19 |
| Cortese et al. (2023) | Multicentre | 232 (DCB 118 / DES 114) | 83 / 35 vs. 87 / 27 | 64.0 ± 23.7 vs. 66.0 ± 23.7 | De novo small vessel disease (reference diameter 2.0–2.75 mm; stable or acute coronary syndrome) | DCB-PTX: Elutax SV paclitaxel-coated balloon (≥ 30 s expansion) | EES: Xience everolimus-eluting stent | 6 months | TLR |  |  | 9/102 |
| Ardissino et al. (2004) | Italy | 257 (SES 129 / BMS 128) | 99/30 vs. 85/43 | 63.2 ± 11.5 vs. 63.7 ± 10.9 | Previously untreated atherosclerotic lesions, small vessels (RVD ≤ 2.75 mm); ACS, stable angina, or silent ischemia | SES: Sirolimus-Eluting Stent | BMS: Bare-Metal Stent | 8 months | TLR, BR, MI | 12/123 | 60/113 | 9/129 |
| Tsuchikane et al. (2004) | Japan | 106 (BMS 50 / BA + CIL 56) | 39/11 vs. 44/12 | 64 ± 8 vs. 66 ± 7 | De novo small vessel coronary artery disease (reference diameter < 3.0 mm) | BMS: Bare-Metal Stent | POBA：Balloon Angioplasty | 6 months | TLR, BR, MI | 12/49 | 7/56 | 11/50 |
| Braun et al. (2007) | Multicentre | 222 (CS 116 / POBA 106) | 44/12 vs. 39/11 | 65.0 ± 9.5 vs. 63.3 ± 9.0 | De novo small vessel disease (reference diameter ≤ 2.8 mm; stable or unstable angina) | CCS: SYNCRO™ Carbon Coated Stent In situ Implantation (Pre-expanded or Direct Implant) | POBA: 2.5 mm conventional balloon dilatation (allows transition to the same CS in case of residual stenosis or rupture) | 6 months | TLR, BR, MI | 12/101 | 28/86 | 12/103 |
| Tian et al. (2020) | China | 230 (DCB 116 / DES 114) | 77 / 39 vs. 88 / 26 | 60.1 ± 10.5 vs. 60.5 ± 10.8 | De novo small vessel disease (RVD 2.25–2.75 mm) | DCB-PTX: RESTORE drug-coated balloon (paclitaxel; Cardionovum) | EES: RESOLUTE Integrity everolimus-eluting stent (Medtronic) | 9 months | TLR, BR, MI | 2/29 | 9/71 | 6/115 |
| Funatsu et al. (2017) | Japan | 133 (DCB 92 / POBA 41) | 72/20 vs. 28/13 | 68 ± 10 vs. 69 ± 11 | De novo small vessel coronary artery disease (reference diameter 2.0–2.75 mm; stable/unstable angina or silent ischemia) | DCB-PTX: SeQuent Please® drug-coated balloon (paclitaxel–iopromide 3 µg/mm², inflation ≥7 atm ≥30 s) | POBA: SeQuent conventional uncoated balloon; allows rescue BMS if necessary | 6 months | TLR, BR, MI | 13/98 | 17/40 | 2/88 |
| Umeda et al. (2004) | Japan | 263 (GPBA 85 / CBA 88 / POBA 90) | 60 / 25 vs. 64 / 24 vs. 66 / 24 | 65.9 ± 10.1 vs 64.9 ± 9.0 vs 66.6 ± 9.5 | De novo small vessel disease (RVD ≤ 3.0 mm; stable or unstable angina) | (a) GPBA: primary gradual & prolonged balloon angioplasty（cumulative inflation ≥10 min） (b) CBA: cutting balloon angioplasty | POBA: plain old balloon angioplasty | 6 months | TLR, BR, MI | (a) 26/83 (b) 28/85 | 43/85 | (a) 17/83 (b) 17/85 |
| Rodriguez et al. (2005) | Multicentre | 220 (PES 111 / POBA 109) | 81 / 30 vs. 84 / 25 | NA | Diabetic patients with de novo small vessel disease (reference diameter 2.0–<2.9 mm) | PES: Primary stenting with PC-coated Biodivisio SV stent | POBA: Plain old balloon angioplasty (PTCA) with provisional stenting | 6-9 months | TLR, BR, MI | 20/80 | 35/79 | 17/111 |
| De Luca et al. (2006) | Netherlands | 798 (BMS 387 / POBA 411) | 287/100 vs. 305/106 | 61 ± 12 vs. 61 ± 12 | Small vessels in STEMI patients (post-procedural RVD ≤ 3.0 mm) | BMS: Bare-Metal Stent | POBA: plain old balloon angioplasty with provisional stenting | 6 months | TLR, BR, MI | 59/150 | 83/171 | 69/387 |
| Hermiller et al. (2009) | USA | 219 (EES 160 / PES 59) | 97/63 vs. 28/31 | 63.84 ± 10.71 vs. 63.62 ± 10.31 | De novo small vessel disease (RVD 2.34 ± 0.33 vs. 2.36 ± 0.30 mm) | EES: XIENCE V cobalt-chromium everolimus-eluting stent (100 µg/cm²) | PES: TAXUS Express paclitaxel-eluting stent | 9 months | TLR, BR, MI | 3/74 | 5/24 | 2/154 |
| Hanekamp et al. (2004) | Multicentre | 496 (BMS 250 / POBA 246) | 160/90 vs. 175/71 | 61 ± 9 vs. 61 ± 10 | De novo small vessel disease (reference diameter 2.0–3.0 mm) | BMS: Elective silicon-carbide–coated Tenax™ stent (Biotronik) | POBA: Plain old balloon angioplasty with provisional stenting | 6 months | BR, MI | 42/166 | 40/189 |  |
| Ortolani et al. (2005) | Italy | 74 (SES 29 / BMS 45) | 20 / 9 vs. 32 / 13 | 66 ± 11 vs. 65 ± 9 | De novo small vessel disease (reference diameter ≤ 2.75 mm) | SES: Sirolimus-Eluting Stent (Cypher™, Cordis) | BMS: Bare-Metal Stent (Bx Sonic™, Cordis) | 8 ± 0.5 months | TLR, BR, MI | 7/28 | 26/41 | 5/29 |
| Rodriguez et al. (2005) | Multicentre | 246 (BMS 124 / POBA 122) | 100 / 24 vs. 91 / 31 | NA | De novo small vessel disease (RVD 2.0–2.9 mm; ACS or stable angina) | BMS: BiodivYsio SV phosphatidylcholine-coated bare metal stent | POBA: POBA with provisional stenting | 9 months | TLR, BR, MI | 23/116 | 35/104 | 1/124 |
| Naganuma et al. (2015) | Italy | 182 (DCB 90 / PES 92) | 72 / 18 vs. 71 / 21 | 64.8 ± 8.5 vs. 66.4 ± 9.0 | De novo small vessel disease (reference diameter < 2.8 mm) | DCB-PTX: IN.PACT Falcon™ paclitaxel drug-coated balloon + on-demand bare metal stent | PES: Taxus Libertè® Paclitaxel-Eluting Stent | 6 months | TLR |  |  | 6/90 |
| Schampaert et al. (2004) | Canada | 100 (SES 50/ BMS 50) | 35/15 vs. 34/16 | 60.3 ± 10.6 vs. 60.7 ± 9.1 | De novo long lesion small vessel CAD (lesion length 15–32 mm; RVD 2.5–3.0 mm) | SES: Cypher™, Bx-VELOCITY platform; non-degradable polymer; drug loading approximately 140 µg/cm²; 80% release in 30 days | BMS: same platform, uncoated Bx-VELOCITY | 9 months | TLR, BR, MI | 1/44 | 23/44 | 2/50 |
| Kereiakes et al. (2017) | USA | 2008 (BVS 1322/ EES 686) | 934/388 vs. 481/205 | 63.5±10.6 vs. 63.6±10.3 | Small vessel coronary artery disease (vessel diameter ≤3.0 mm) | BVS-EES: Everolimus-Eluting Bioresorbable Scaffold | EES: cobalt-chromium everolimus-eluting stents (Xience EES) | 12 months | TLR |  |  | 92/1322 |
| Tang et al. (2018) | China | 230 (DCB 116/DES 114) | 77/39 vs. 88/26 | 60.1 ± 10.5 vs. 60.5 ± 10.8 | Coronary small-vessel disease (RVD ≤ 2.75 mm; de novo lesion) | DCB-PTX: Restore paclitaxel-coated balloon (Cardionovum, Bonn, Germany) with mandatory lesion pre-dilation; bailout bare-metal stent if severe dissection (class D–F) or residual stenosis > 30% post-DCB | ZES: RESOLUTE Integrity zotarolimus-eluting stent with operator-discretion pre- or post-dilation | 9 months | TLR, BR, MI | 11/100 | 8/93 | 5/114 |
| Buiten et al. (2020) | Multicentre | 898 (ZES 454/SES 444) | 344/110 vs. 330/114 | 65.1 ± 10.9 vs. 64.0 ± 11.0 | Coronary small-vessel disease (RVD <2.5 mm; de novo or restenotic lesions) | ZES: Zotarolimus-Eluting Stent | SES: Ultrathin-strut biodegradable-polymer Orsiro sirolimus-eluting stent (SES), with strut thickness 60 µm for stents ≤3.0 mm and 80 µm for stents ≥3.5 mm, eluting sirolimus for 4 months | 24 months | TLR |  |  | 18/454 |
| Schofer et al. (2003) | Multicentre | 352 (SES 175/ BMS 177) | 123/52 vs. 126/51 | 62.0 ± 11.4 vs. 62.6 ± 10.3 | Coronary small-vessel disease (RVD 2.5–3.0 mm; de novo lesion) | SES: Sirolimus-eluting Bx Velocity balloon-expandable stent, with 5 µm coating (33% sirolimus, 67% non-erodable polymer, 140 µg/cm² sirolimus), releasing ~80% sirolimus within 30 days | BMS: Bare-metal Bx Velocity balloon-expandable stent, uncoated, identical architecture to sirolimus-eluting stent | 9 months | TLR, BR, MI | 9/152 | 66/156 | 7/175 |
| Jeger et al. (2018) | Switzerland, Germany | 758 (DCB 382/DES 376) | 290/92 vs. 285/91 | 67.8 ± 10.5 vs. 68.0 ± 10.2 | Coronary small-vessel disease (RVD <3.0 mm; de novo lesion) | DCB-PTX: Paclitaxel-coated balloon (SeQuent Please, B Braun Melsungen AG), inflated at nominal pressure for ≥30 s after successful predilatation (no higher-grade dissections, TIMI flow ≥2, residual stenosis ≤30%); additional spot stenting if flow-limiting dissections or >30% residual stenosis | EES: Second-generation DES (everolimus-eluting Xience, Abbott Vascular, or paclitaxel-eluting Taxus Element, Boston Scientific), per standard PCI guidelines | 12 months | TLR |  |  | 13/382 |
| Savage et al. (1998) | USA, Canada, Japan | 331 (BMS 163/GPBA 168) | 121/42 vs. 114/54 | 59 ± 10 vs. 61 ± 11 | Coronary small-vessel disease (RVD <3.0 mm; de novo lesion) | BMS: Elective placement of Palmaz-Schatz bare-metal stent, balloon-expandable, deployed at 10–16 atmospheres, up to two stents to cover lesion with 2–4 mm overlap if needed | GPBA: Balloon angioplasty, standard interventional techniques, with optional predilation at investigator’s discretion | 6 months | TLR, BR, MI | 47/139 | 66/121 | 26/163 |
| Park et al. (2000) | South Korea | 120 (BMS 60/GPBA 60) | 37/23 vs. 39/21 | 60.2 ± 7.5 vs. 61.5 ± 8.4 | Coronary small-vessel disease (RVD <3.0 mm; de novo lesion) | BMS: Elective placement of 7-cell NIR stent (Boston Scientific), balloon-expandable, deployed at ≥10 atmospheres, with adjunct high-pressure balloon dilation if needed for angiographic optimization | GPBA: Optimal balloon angioplasty, moderately high-pressure inflation (≥10 atmospheres), aiming for diameter stenosis ≤30% and no major dissection (NHLBI types C–F); crossover to stenting allowed for suboptimal results (>30% stenosis) or major dissection | 6 months | TLR, BR, MI | 20/56 | 17/55 | 5/60 |
| Moer et al. (2002) | Norway, Sweden | 145 (BMS 74/GPBA 71) | 42/32 vs. 52/19 | 63.1 ± 11.2 vs. 62.7 ± 10.1 | Coronary small-vessel disease (RVD 2.1–3.0 mm; de novo lesion) | BMS: Elective placement of Hepamed-coated beStent (Medtronic InStent), balloon-expandable, single 15-mm stent, deployed per standard interventional techniques | GPBA: Percutaneous transluminal coronary angioplasty (PTCA), standard interventional techniques, with optional predilation per local standards | 6 months | TLR, BR, MI | 7/72 | 13/69 | 5/74 |
| Menozzi et al. (2009) | Italy | 257 (SES 129/BMS 128) | 91/38 vs. 89/39 | 64.2 ± 10.1 vs. 64.7 ± 9.8 | Coronary small-vessel disease (RVD 2.25–2.75 mm; de novo lesion) | SES: Sirolimus-Eluting Stent (Cypher™, Cordis), balloon-expandable, with sirolimus coating (non-degradable polymer, drug loading approximately 140 µg/cm², ~80% release in 30 days) | BMS: Bare-Metal Stent (Bx Sonic, Cordis), balloon-expandable, uncoated, identical structure to SES | 8 months | TLR, BR, MI | 9/114 | 39/114 | 10/127 |
| Hausleiter et al. (2004) | Germany | 502 (BMS 253/GPBA 249) | 193/60 vs. 185/64 | 65.3 ± 10.2 vs. 65.7 ± 10.5 | Coronary small-vessel disease (RVD ≤2.5 mm; de novo lesion) | BMS: Phosphorylcholine-coated BiodivYsio SV stent (Abbott Vascular Devices), balloon-expandable, strut thickness ~90 µm, designed for 2.0–2.75 mm vessels, deployed to achieve <30% residual stenosis and TIMI flow grade 3 | GPBA: Percutaneous transluminal coronary angioplasty (PTCA), standard interventional techniques, aiming for <30% residual stenosis and TIMI flow grade 3; crossover to stenting allowed for suboptimal results (TIMI flow <3, large residual dissection ≥5 mm, or residual stenosis >30%) | 6-8 months | TLR, BR, MI | 79/204 | 69/201 | 51/253 |
| Cortese et al. (2010) | Italy | 57 (DCB 28/PES 29) | 20/8 vs. 24/5 | 65.2 ± 11.4 vs. 64.8 ± 10.9 | Coronary small-vessel disease (RVD ≤2.75 mm; de novo lesion) | DCB-BMS: Dior paclitaxel-coated balloon (Eurocor, Bonn, Germany), nanoporous balloon with paclitaxel microcrystals (3 µg/mm²), inflation 45 s repeated twice, 8-16 atm; bailout BMS if unsatisfactory (persistent dissection >A or TIMI <3) | PES: Taxus Libertè paclitaxel-eluting stent (Boston Scientific), predilatation recommended, inflation 20-30 s, postdilated with non-compliant balloon | 9 months | TLR, BR, MI | 9/28 | 3/29 | 10/28 |
| Kastrati et al. (2000) | Germany | 404 (BMS 204/GPBA 200) | 149/55 vs. 147/53 | 64.8 ± 10.3 vs. 65.2 ± 10.6 | Coronary small-vessel disease (RVD 2.0–2.8 mm; de novo lesion) | BMS: Elective placement of MULTI-LINK stent (Guidant, Advanced Cardiovascular Systems), premounted on ≥2.5 mm balloons, deployed to achieve <30% residual stenosis and TIMI flow grade 3 | GPBA: Percutaneous transluminal coronary angioplasty (PTCA), standard methods, aiming for <30% residual stenosis and TIMI flow grade 3; crossover to stenting allowed for large dissections (≥5 mm) or TIMI flow <3 | 7 months | TLR, BR, MI | 61/170 | 62/165 | 41/204 |
| Iglesias et al. (2019) | Switzerland | 1234 (SES 637/EES 597) | 473/164 vs. 444/153 | 66.5 ± 10.9 vs. 66.7 ± 10.7 | Coronary small-vessel disease (RVD ≤3.0 mm; de novo or restenotic lesions) | SES: Orsiro biodegradable polymer sirolimus-eluting stent (Biotronik), ultrathin-strut (60 µm for stents ≤3 mm), sirolimus dose 1.4 µg/mm², biodegradable PLLA polymer, fully degraded in 12–24 months | EES: Xience durable polymer everolimus-eluting stent (Abbott Vascular), thin-strut (81 µm), everolimus dose 100 µg/cm², fluorinated copolymer, deployed per standard PCI guidelines | 5 years | TLR |  |  | 62/637 |
| Dan et al. (2020) | Multicentre | 1082 (SES 699/EES 383) | 525/174 vs. 287/96 | 65.1 ± 10.8 vs. 65.5 ± 10.6 | Coronary small-vessel disease (RVD ≤2.75 mm; de novo or restenotic lesions) | SES: Orsiro bioresorbable-polymer sirolimus-eluting stent (Biotronik), ultrathin-strut (60 µm for stents ≤3 mm), sirolimus dose 1.4 µg/mm², biodegradable PLLA polymer, fully degraded in 12–24 months, deployed per standard PCI guidelines | EES: Xience durable-polymer everolimus-eluting stent (Abbott Vascular), thin-strut, everolimus dose , fluorinated copolymer, deployed per standard PCI guidelines | 12 months | TLR |  |  | 35/699 |
| Koning et al. (2001) | France | 381 (BMS 192/GPBA 189) | 146/46 vs. 142/47 | 62.7 ± 10.5 vs. 63.1 ± 10.8 | Coronary small-vessel disease (RVD ≤3.0 mm; de novo lesion) | BMS: Elective placement of beStent (Medtronic), balloon-expandable, single stent crimped over a ≥2.5-mm or noncompliant 2.75-mm balloon, deployed to cover lesion ≤15 mm | GPBA: Standard balloon angioplasty, using noncompliant balloons (2.5–2.75 mm), aiming for optimal angiographic result (<30% residual stenosis, no flow-limiting dissection) | 6 months | TLR, BR, MI | 37/175 | 81/172 | 25/192 |
| Jeger et al. (2016) | Switzerland | 191 (ZES 91/PES 100) | 69/22 vs. 75/25 | 67.3 ± 9.8 vs. 66.8 ± 10.2 | Coronary small-vessel disease (RVD ≤2.75 mm; de novo or restenotic lesions) | ZES: Endeavor Sprint zotarolimus-eluting stent (Medtronic), thin-strut (91 µm), zotarolimus dose 10 µg/mm, phosphorylcholine polymer, deployed per standard PCI guidelines | PES: Taxus Liberté paclitaxel-eluting stent (Boston Scientific), thin-strut (97 µm), paclitaxel dose 1 µg/mm², Translute polymer, deployed per standard PCI guidelines | 2 years | TLR |  |  | 6/91 |
| Colombo et al. (2015) | Italy | 182 (DCB 90/PES 92) | 67/23 vs. 68/24 | 65.4 ± 10.2 vs. 65.8 ± 9.7 | Coronary small-vessel disease (RVD <2.8 mm; de novo lesion) | DCB-BMS: In.Pact Falcon paclitaxel-coated balloon (Medtronic), drug dose 3 µg/mm², inflation 30–60 s after predilatation; provisional bare-metal stenting if residual stenosis >30% or flow-limiting dissection | PES: Taxus Liberté paclitaxel-eluting stent (Boston Scientific), thin-strut (97 µm), paclitaxel dose 1 µg/mm², Translute polymer, deployed per standard PCI guidelines | 3 years | TLR, BR, MI | 12/83 | 11/85 | 9/90 |
| Xu et al. (2022) | China | 212 (DCB 105/GPBA 107) | 78/27 vs. 80/27 | 62.1 ± 10.3 vs. 61.8 ± 9.9 | Coronary small-vessel disease (RVD 2.0–2.75 mm; de novo lesion) | DCB-BMS: Biolimus-coated balloon (Biosensor International), drug dose 0.6 µg/mm², inflation 30–60 s after predilatation; provisional bare-metal stenting if residual stenosis >30% or flow-limiting dissection | GPBA: Plain old balloon angioplasty (POBA), standard interventional techniques, using non-coated balloons, aiming for <30% residual stenosis and no flow-limiting dissection | 12 months | TLR, BR, MI | 10/96 | 20/98 | 5/105 |

Note: DCB Drug-Coated Balloon, DCB-PTX Paclitaxel-Coated Drug-Coated Balloon, DES Drug-Eluting Stent, EES Everolimus-Eluting Stent, ZES Zotarolimus-Eluting Stent, SES Sirolimus-Eluting Stent, PES Paclitaxel-Eluting Stent, BMS Bare-Metal Stent, BVS Bioresorbable Vascular Scaffold, BVS-EES Everolimus-Eluting Bioresorbable Scaffold, CCS Carbon-Coated Stent, POBA Plain Old Balloon Angioplasty, BP Balloon Predilation, BA+CIL Balloon Angioplasty plus Cilostazol, CBA Cutting Balloon Angioplasty, GPBA Gradual and Prolonged Balloon Angioplasty, RA Rotational Atherectomy, TLR Target Lesion Revascularization, BR Binary Restenosis, MI Myocardial Infarction, CAD Coronary Artery Disease, ACS Acute Coronary Syndrome, STEMI ST-Elevation Myocardial Infarction, RVD Reference Vessel Diameter, LAD Left Anterior Descending artery.

# Table S3: Risk of Bias

| **Author** | **Bias arising from the randomization process** | **Bias due to deviations from intended intervention** | **Bias due to missing outcome data** | **Bias in measurement of the outcome** | **Bias in selection of the reported result** | **Overall** |
| --- | --- | --- | --- | --- | --- | --- |
| Fahrni et al. (2020a) | Some concerns | Some concerns | Some concerns | Low | Low | Some concerns |
| Giannini et al. (2017) | Low | Some concerns | Low | Low | Low | Some concerns |
| Jeger et al. (2020b) | Low | Low | Low | Low | Low | Low |
| Kwon et al. (2003) | Low | Low | Low | Low | Low | Low |
| Liu et al. (2024) | Low | Low | Low | Low | Low | Low |
| Kawai et al. (2021) | Low | Some concerns | Some concerns | Low | Low | Some concerns |
| Cortese et al. (2023) | Low | Some concerns | Low | Low | Low | Some concerns |
| Ardissino et al. (2004) | Low | Some concerns | Low | Low | Low | Some concerns |
| Tsuchikane et al. (2004) | Low | Low | Low | Low | Low | Low |
| Braun et al. (2007) | Low | Some concerns | Some concerns | Low | Low | Some concerns |
| Tian et al. (2020) | Some concerns | Low | Some concerns | Low | Low | Some concerns |
| Funatsu et al. (2017) | Some concerns | Low | Some concerns | Low | Low | Some concerns |
| Umeda et al. (2004) | Low | Low | Some concerns | Low | Low | Some concerns |
| Rodriguez et al. (2005) | Low | Low | Low | Low | Low | Low |
| De Luca et al. (2006) | Low | Low | Low | Low | Low | Low |
| Hermiller et al. (2009) | Low | Low | Low | Low | Low | Low |
| Hanekamp et al. (2004) | Low | Low | Low | Low | Low | Low |
| Ortolani et al. (2005) | Low | Low | Some concerns | Low | Low | Some concerns |
| Rodriguez et al. (2005) | Low | Low | Low | Low | Low | Low |
| Naganuma et al. (2015) | Low | Low | Some concerns | Low | Low | Some concerns |
| Schampaert et al. (2004) | Low | Some concerns | Some concerns | Low | Low | Some concerns |
| Kereiakes et al. (2017) | Low | Low | Low | Low | Low | Low |
| Tang et al. (2018) | Low | Low | Low | Low | Low | Low |
| Buiten et al. (2020) | Low | Low | Low | Low | Low | Low |
| Schofer et al. (2003) | Low | Some concerns | Low | Low | Low | Some concerns |
| Jeger et al. (2018) | Low | Low | Some concerns | Low | Low | Some concerns |
| Savage et al. (1998) | Low | Some concerns | Some concerns | Low | Low | Some concerns |
| Park et al. (2000) | Low | Low | Low | Low | Low | Low |
| Moer et al. (2002) | Low | Low | Low | Low | Low | Low |
| Menozzi et al. (2009) | Low | Low | Low | Low | Low | Low |
| Hausleiter et al. (2004) | Low | Low | Low | Low | Low | Low |
| Cortese et al. (2010) | Low | Low | Some concerns | Low | Low | Some concerns |
| Kastrati et al. (2000) | Low | Low | Low | Low | Low | Low |
| Iglesias et al. (2019) | Low | Low | Some concerns | Low | Low | Some concerns |
| Dan et al. (2020) | Some concerns | Low | Some concerns | Low | Low | Some concerns |
| Koning et al. (2001) | Low | Low | Some concerns | Low | Low | Some concerns |
| Jeger et al. (2016) | Low | Low | Low | Low | Low | Low |
| Colombo et al. (2015) | Low | Low | Low | Low | Low | Low |
| Xu et al. (2022) | Low | Low | Low | Low | Low | Low |

# **Table S4: Meta-regression with strut thickness**

| **Outcome** | **Coefficient β (per 10 μm)** | **OR per 10 μm** | **95% CI (OR)** | **p-value** | **τ² (unadjusted)** | **τ² (adjusted for thickness)** | **Δτ²** |
| --- | --- | --- | --- | --- | --- | --- | --- |
| TLR | 0.068 | 1.07 | 1.02-1.12 | 0.006 | 0.12 | 0.09 | -0.03 |
| BR | 0.086 | 1.09 | 1.03-1.15 | 0.002 | 0.18 | 0.13 | -0.05 |
| MI | 0.037 | 1.04 | 0.99-1.09 | 0.11 | 0.1 | 0.09 | -0.01 |

# Figure S1: Funnel Plots of Publication Bias


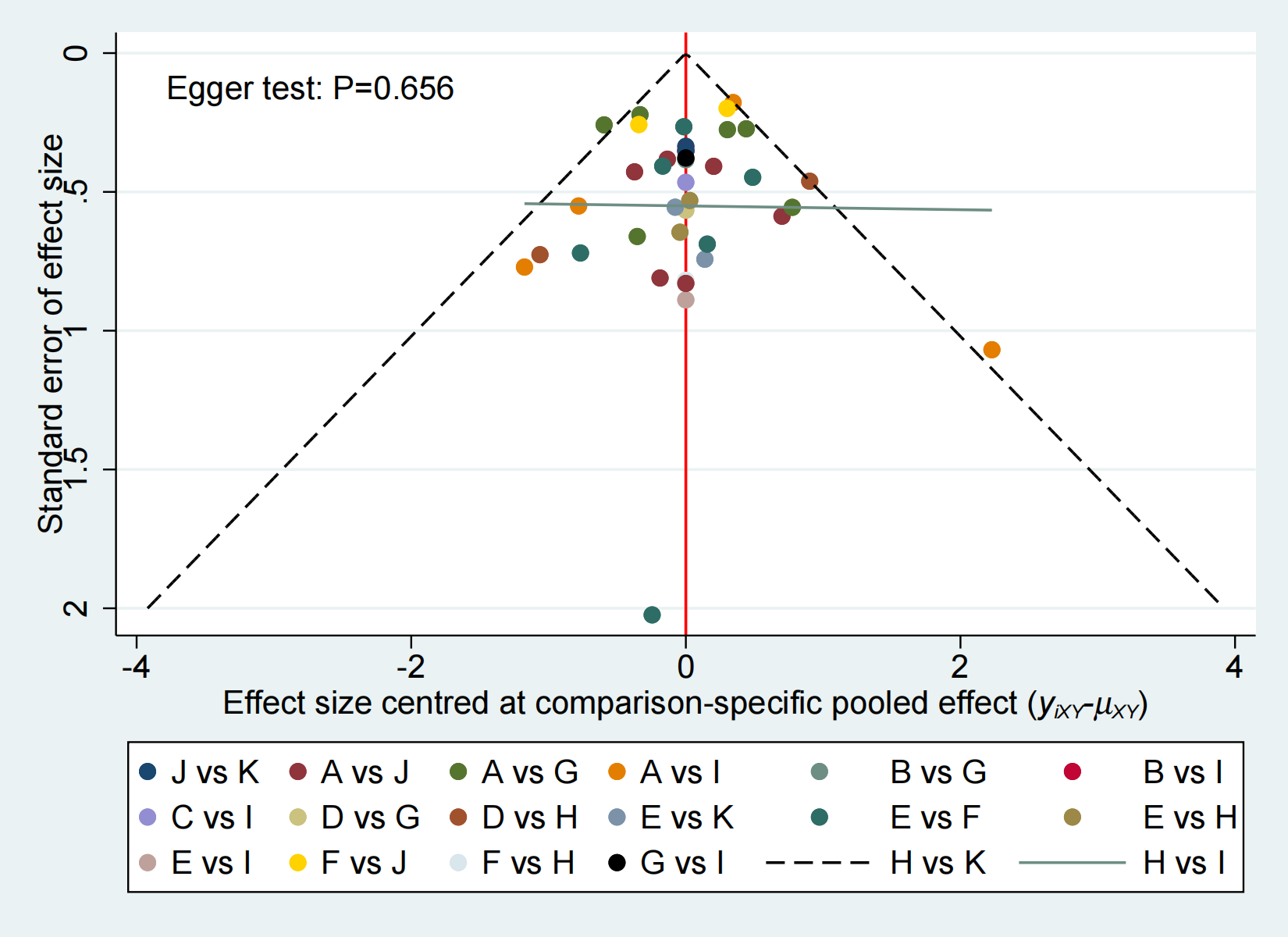


Figure S1.1 The funnel plot of TLR. The result of Egger test showed the p=0.656.


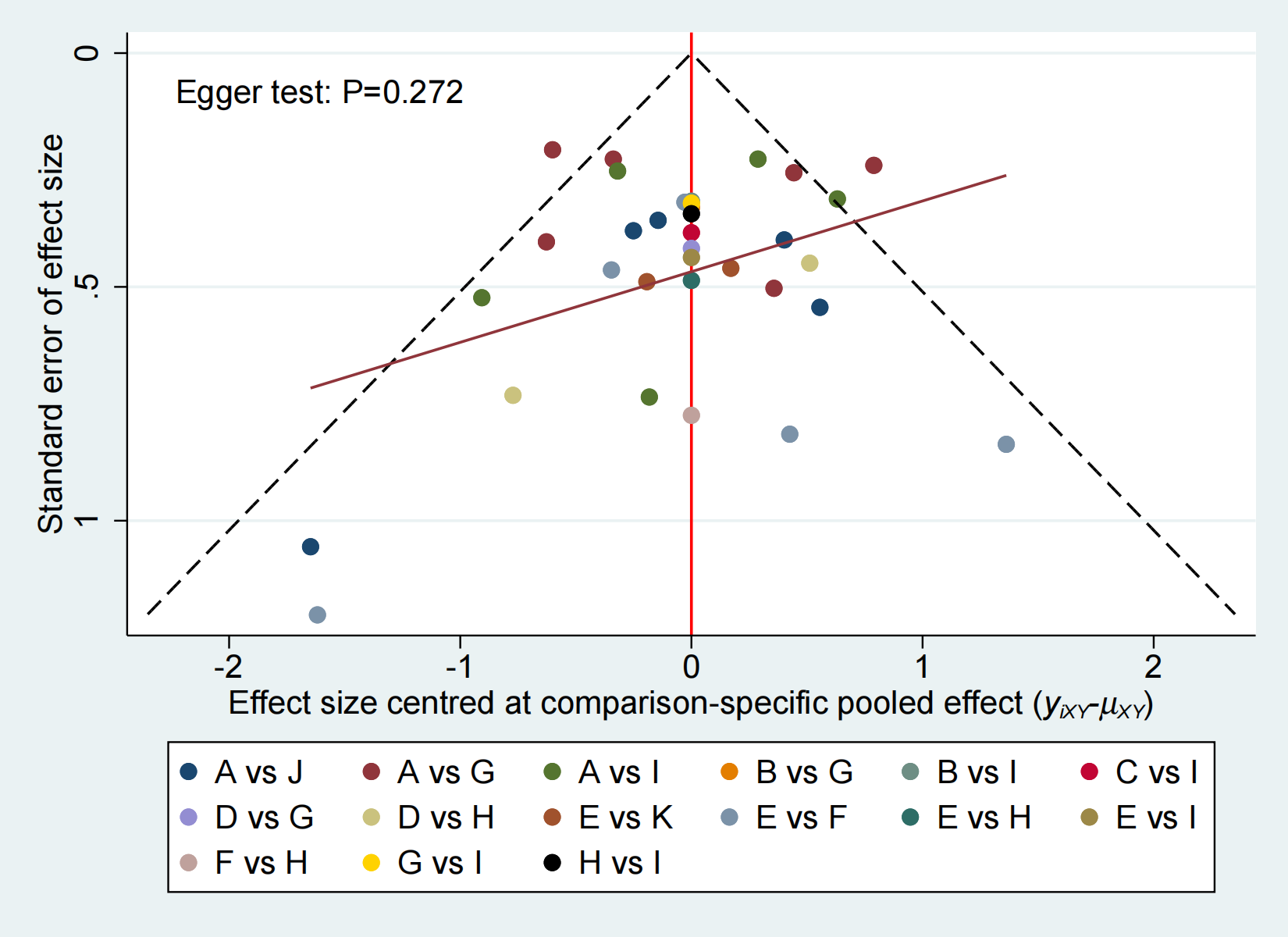


Figure S1.2 The funnel plot of BR. The result of Egger test showed the p=0.272.


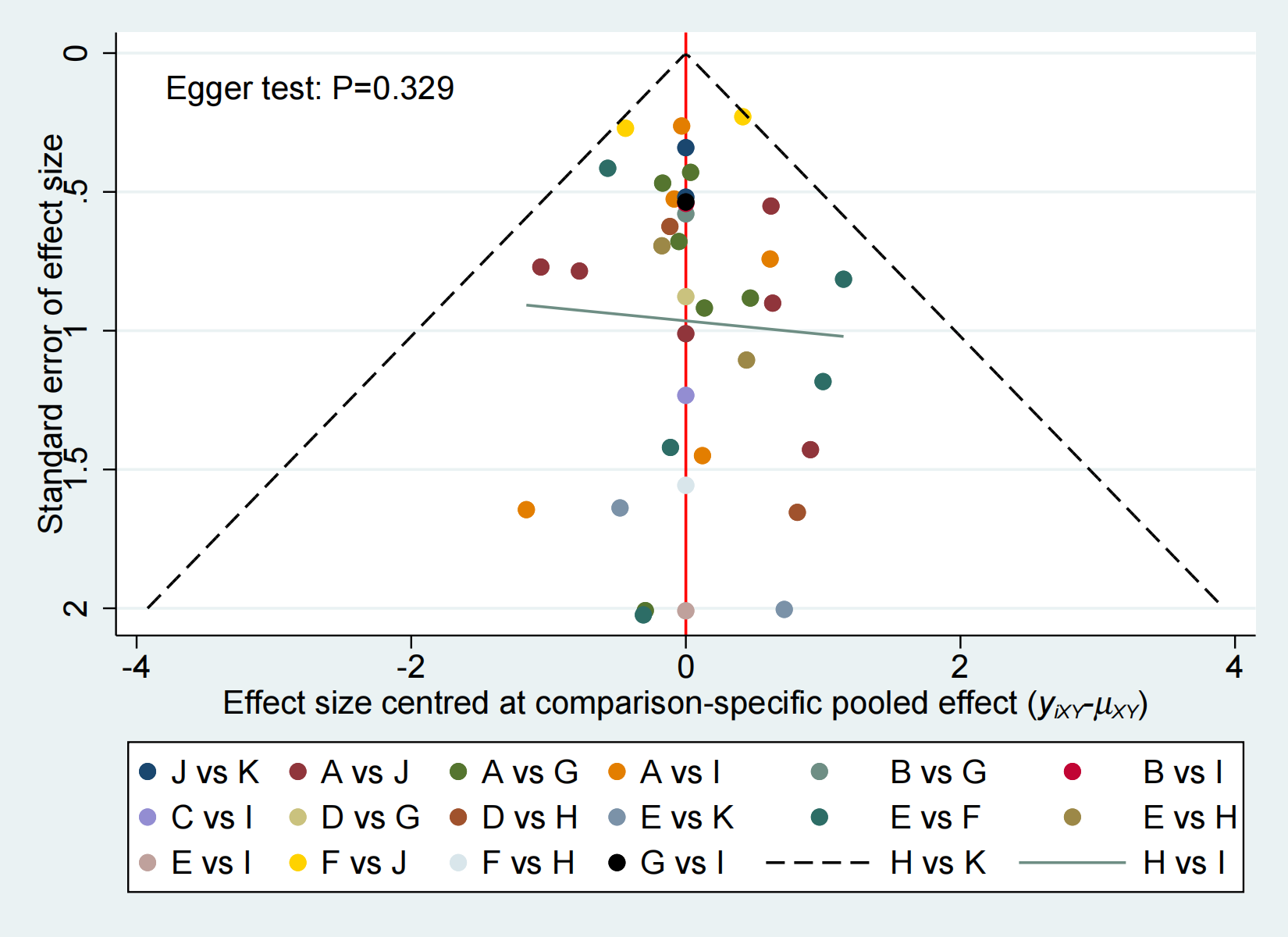


Figure S1.3 The funnel plot of MI. The result of Egger test showed the p=0.329.

# **Figure S2: SUCRA probability ranking plot**


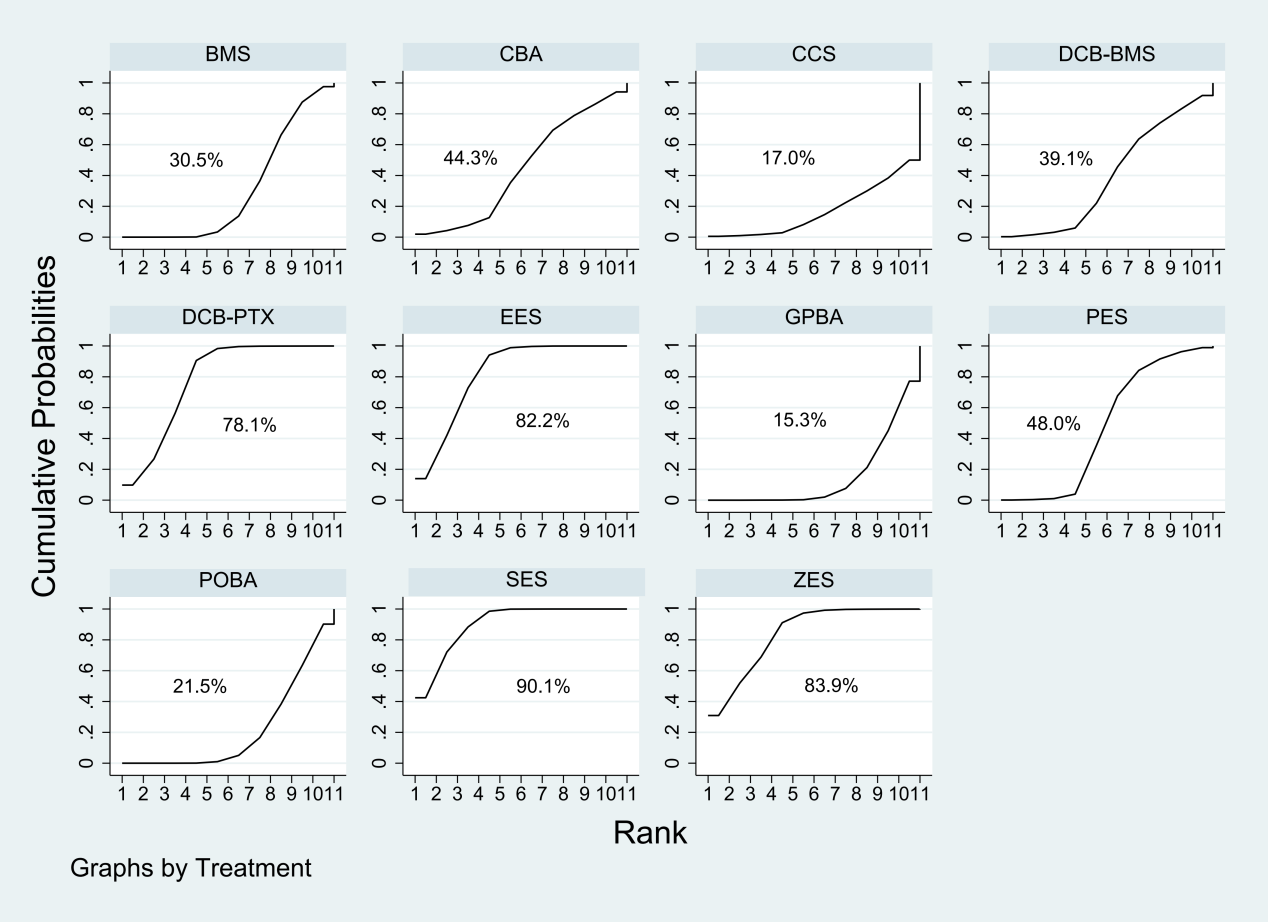


Figure S2.1 The SUCRA probability ranking plot of TLR.


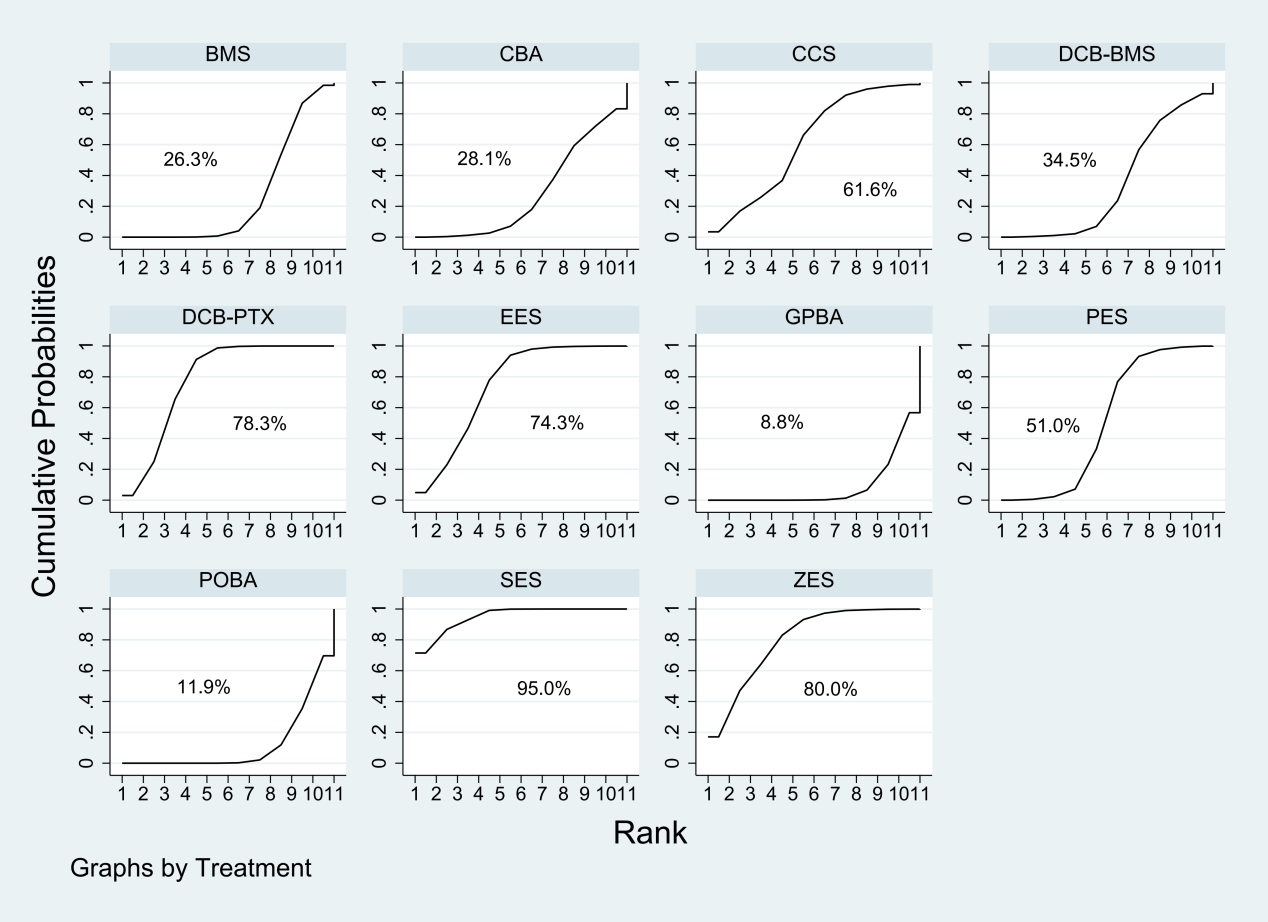


Figure S2.2 The SUCRA probability ranking plot of BR.


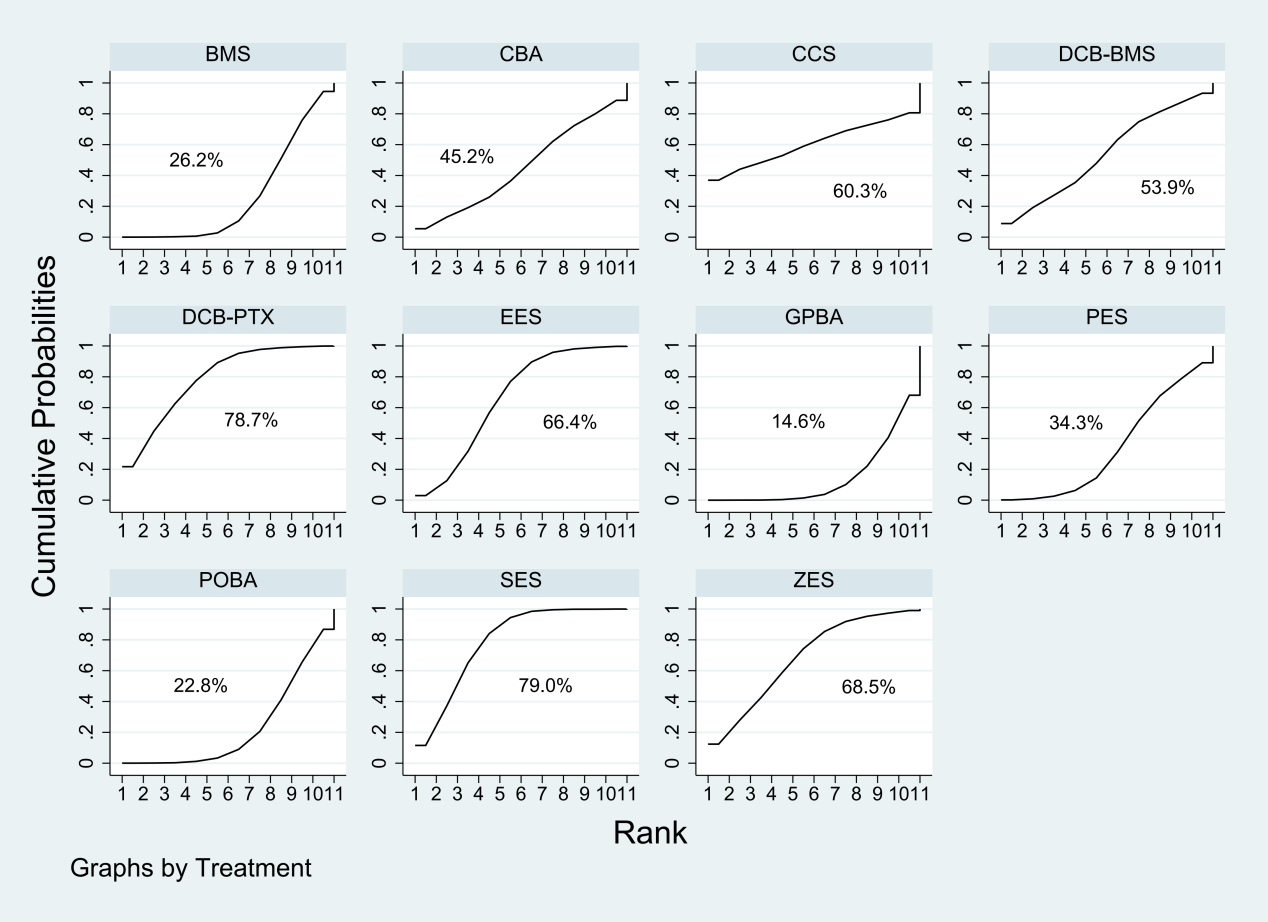


Figure S2.3 The SUCRA probability ranking plot of MI.
